# A Wonderful Medium

**Published:** 1887-06

**Authors:** 


### A WONDERFUL MEDIUM.

Fanny Treiber, the daughter of a Minneapolis, Minn., washerwoman, has developed wonderful powers as a medium. She is nine years old, but is very ignorant, never having received a common school education, and is neither able to read nor write. A few days ago the girl told her mother about "having funny dreams," in which she discoursed with several dead relatives. Shortly after the girl took a slate and pencil, of an elder brother and began writing in a clear legible hand, what seemed to be messages from the people who had departed from this life. The writing was peculiar in form, being right to left instead of left to right, and was read by the little one's mother by holding the slate before a looking-glass.

A day or two ago a prominent business man was called upon by the mother, to see what he thought of the matter, she being mystified and alarmed. It was only a moment after going to see the girl and talking with her that she passed into the peculiar condition attending the demonstrations known as the "trance state," and wrote him a message to all intents from his wife, who has been dead some time, signing her name. The communication carried information upon certain domestic affairs that the gentleman says, no one but his wife and himself had the slightest knowledge of, least of all the ignorant little girl who could not ordinarily write her own name, and whom he never saw or heard of before A public test of the girl's powers will be made.—*Dayton* (*Ohio*) *Journal.*

---

William H. Vanderbilt gave $500,000 to the college of Physicians and Surgeons here, to help a system of medicine which deals in many harsh and fierce remedies, and which does not pretend to be founded on any definite scientific principles. If the College of Magnetics, presided over by the eminent scientist, Dr E. D. Babbitt, had a hundredth part of this amount, he would be able to bring out his discoveries in the basic principles of all science, in good style before the world, and to proclaim to millions of people through the press these better laws of life.

